# Neuroanesthesia management of neurosurgery of brain stem tumor requiring neurophysiology monitoring in an iMRI OT setting

**DOI:** 10.4103/1658-354X.57877

**Published:** 2009

**Authors:** Abdulrahmam J. Sabbagh, Mahmoud Al-Yamany, Reem F. Bunyan, Mohamad S. M. Takrouri, Sabry Mohammed Radwan

**Affiliations:** *Department of Neurosurgery, King Fahd Medical City, PO Box - 59046, Riyadh - 115 25, Kingdom of Saudi Arabia*; 1 *Department of Neurology and Neurophysiology, King Fahd Medical City, PO Box - 59046, Riyadh - 115 25, Kingdom of Saudi Arabia*; 2 *Department of Anesthesia, Neuroscience Center (020007), King Fahd Medical City, PO Box - 59046, Riyadh - 115 25, Saudi Arabia*

**Keywords:** *Brain stem*, *brainSUITE^®^ intra-operative magnetic resonance imaging operating theater*, *evoked responses/potentials*, *neuromonitoring*, *neuroanesthesia*, *Motor EP: Recording from cranial nerve supplied muscle*, *Sensory EP: Medial/tibial*

## Abstract

This report describes a rare case of ventrally exophytic pontine glioma describing operative and neuroanesthesia management. The combination of intraoperative neuromonitoring was used. It constituted: Brain stem evoked responses/potentials, Motor EP: recording from cranial nerve supplied muscle, and Sensory EP: Medial/tibial. Excision of the tumor was done with intra-operative magnatic resonance imaging (iMRI), which is considered a new modality.

## INTRODUCTION

This case report describes the operative and neuroanesthesia management of a rare case of ventrally exophytic pontine glioma. It was guided by a new intraoperative neuromonitoring, which demanded some modifications in anesthesia management, such as, elimination of muscle relaxants and certain drugs used in neuroanesthesia.

Also the presence in the special environment of the *brainSUITE*^®^ intra-operative magnetic resonance imaging operating theater (iMRI OT) demands magnetic precautions and close monitoring of the patient's welfare.

## CASE REPORT

A six-year-old healthy boy, who was born at term, with normal development, presented to the emergency room with a headache and some unsteady gait. The parents mentioned that he had an abnormal eye movement on the right side. On examination, this child showed evidence of partial sixth nerve palsy on the right side and some ataxia while walking, but the rest of his neurological examination was normal. He had no papilledema.

**Figure 1(a-c):**
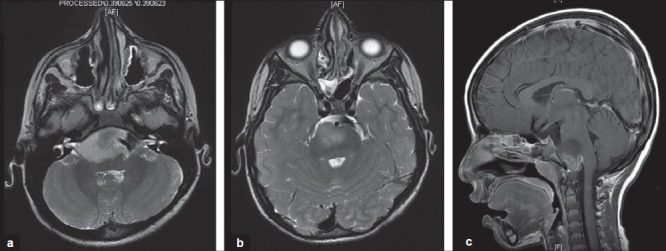
Three MRI of the patient under study showing lesion occupied most of the anterior pons and that was ventrally exophytic going down; hypodense on CT and hypo intense on MRI. The lesion surrounded completely incased a part of the vertebral junction and had component that enhanced with gal linear infusion. No part of these lesions was hypodense on CT and hypo intense on MRI.

The CT scan and MRI showed a lesion occupying most of the anterior pons. It was ventrally exophytic, extending inferiorly; hypodense on CT brain. The lesion surrounded completely, encased a part of the vertebral-basilar junction, and had a component that was enhanced with gadolinium infusion [Figure 1].

Discussion between the pediatric neurosurgeon (AJS)and skull base surgeon (MY) addressed the possibility of approaching this tumor through several options[1–10] namely:

- transorally
- transnasally
- subtemporally
- transtentorially
- far lateral retrosigmoid approach
- a modified trans petrosal presigmoid approach

The decision was to take option (f) with the help of neurophysiology monitoring and iMRI; two surgical stages were planned the first was to do modified petresectomy and the second to resect the tumor.

On the day of the surgery the neurophysiologists set up electrodes necessary for neuromonitoring. Electrocardiogram (ECG), non-invasive blood pressure (NIBP), pulse oximetry, and core temperature were monitored during the operation. After pre-oxygenation, anesthesia was induced with the Propfol 3 mg/kg (Body weight 28 kg) Fentanyl 3 μg/kg Rocuoronium 0.6 mg/kg sequence, followed by endotracheal intubation and artificial ventilation, using air: Oxygen mixture carrying sevoflurane 2.5% was reduced to 0.6-2% through the procedure. Invasive monitoring was installed using an aseptic technique. The anesthesia was then maintained with continuous infusions of Propofol 5 mg/kg/h and Fentanyl at the rate of 2 μg/kg/h.

The surgical technique included insertion of a lumbar drain, the use of 3D reconstruction navigation CT scan images, integrated with navigation MRI sequences at T_1_, which were performed in order to get a pre-sigmoid approach to the brain stem. During this procedure, motor and sensory evoke potentials and direct stimulation were used to monitor the facial and trigeminal nerves and the brain stem. On coming very close to the facial canal, the semicircular canals, and the inner ear, the drilling was stopped; that was done as the first stage. The skin was closed and the patient was sent to the Intensive Care Unit (ICU) after extubation. The next day, the second stage was done in the *brainSUITE*^®^, with intraoperative MRI facility. Neuromonitoring with sensory and motor evoked potentials and direct stimulation was performed.

The dura was opened pre-sigmoidally and the cerebellum was retracted posteriorly, and a very small and tight corridor was maintained. It was performed after completing a pre-op MRI and navigation using T_2_ and T_1_, with contrast integrated neuronavigation. The cranial nerves V, VII, VIII, IX, X, and XI were seen laterally draping the tumor. VI was encased by the tumor. Very carefully the cranial nerves were dissected from the tumor, pushed up and down the tumor. The rest of the tissue was tested by direct stimulation, which was negative. By suctioning and careful easy aspiration 10% of the exophytic part of the tumor was removed. At each stage of tumor removal, direct stimulation was performed and the surgeon came closer to the actual pons, and more sensory and motor evoke potentials were tested. An intraoperative MRI was performed and renewal of registration and navigation was completed, as also further resection of the tumor was performed. The surgeons had excellent neuromonitoring without negative neurological changes and actual improvement of the sensory and motor evoked potentials. The final MRI showed around 70% removal of the tumor, which made the surgeons stop at that point. Postoperatively the lumbar drain that was placed before the first surgery was kept, and placed to drain 10-15 cc of cerebrospinal fluid (CSF) per hour. Intravenous antibiotics were kept on board until the lumbar drain was removed seven days later.

The patient had no new neurological deficits postoperatively, except that the partial sixth nerve palsy that was present preoperatively continued. The patient had a fast recovery with physiotherapy and occupational therapy and was discharged home after having a discussion with the radiation oncologist and the pediatric oncologist, who had decided, on the ground of final pathology grading of highly malignant tumor, to have further radiation. It was decided to follow-up the patient with an MRI in three months and six months time, consecutively.

## DISCUSSION

The initial experience at King Fahad Medical City Neurosciences Center in surgery of modified posterior resection of ventrally exophytic pontine glioma, provided the use new intraoperative neuromonitoring, which constituted: Brain Stem Evoked Responses/potentials (BAER), Motor EP: Recording from cranial nerve supplied muscle (MER), and Sensory EP: Medial/Tibial (SEP).

The anesthesia technique adapted for this new demand of two extensive lengths of surgery was done in two sessions, summing up to 30 hours of surgical work and 48 hours of Surgical Intensive Care Unit (SICU) care.

The neuroanesthesia administered was basically dependent on a combination of Total Intravenous Anesthesia (TIVA) infusion of propofol and fentanyl with fine adjustment of sevoflurane, to allow for a motionless patient and proper neuromonitoring, especially during surgical stimulation, as per the progress in this session. TIVA with propofol and fentanyl or other short-acting narcotics appeared to be well suited and to a lesser extent similar to sevoflurane, during neuromonitoring.[11–22]

The other basic monitoring constituted the measurement of central venous pressure and continuous intra-arterial pressure. The new adaptation of our technique is to allow intraoperative magnetic resonance (iMRI), which neccessitates using long tubing to conduct the respiratory gases and for the inhalational anesthetics to be supplied to the patient, to allow the patient to enter the MRI vault during surgery. Tubing for monitoring and IV fluid management was elongated too. Extra care was given to the special requirement of positioning the head in a shield and under coverage, away from the direct control of the anesthesiologists, during an iMRI session.

## CONCLUSION

This patient was a very rare case of ventrally exophytic pontine tumor, where a subtotal removal was successfully performed. It was through a modified posterior Petrosectomy for a fronto-lateral approach to the pons, with careful and very meticulous dissection of the lower cranial nerves (V, VI, VII, VIII, IX, X, XI, VII).

The time taken for this rare surgical procedure was 30 hours. Prolonged anesthesia time 38 hours in two stages (surgery monitoring of Brain Stem/MRI session). The advantage of prolonged anesthesia using TIVA was quick recovery and allowing evoked potential recording without using muscle relaxants, as also adapting techniques and precautions to avoid complications of long anesthesia in prone position namely: Airway obstruction by mucus plug or kinking of endotracheal tube, pressure sores, difficult extubation, and facial edema.

Team work in the *brainSUITE*^®^ area (surgeon, anesthetist, and neurologist) is essential for the smooth running of this complicated surgery.
